# A crucial new aspect of cardiac morphogenesis: endocardial hematopoiesis

**DOI:** 10.3389/fphys.2024.1525985

**Published:** 2024-12-09

**Authors:** Norika Liu, Atsushi Nakano

**Affiliations:** ^1^ International Research Center for Medical Sciences, Kumamoto University, Kumamoto, Japan; ^2^ Department of Cell Physiology, The Jikei University School of Medicine, Tokyo, Japan; ^3^ Department of Molecular Cell and Developmental Biology, University of California, Los Angeles, Los Angeles, United States; ^4^ David Geffen Department of Medicine, Division of Cardiology, University of California, Los Angeles, Los Angeles, United States; ^5^ Eli and Edythe Broad Center of Regenerative Medicine and Stem Cell Research, University of California, Los Angeles, Los Angeles, United States; ^6^ Molecular Biology Institute, University of California, Los Angeles, Los Angeles, United States

**Keywords:** cardiac development, endocardial cell, hematopoiesis, cardiac cushion, macrophage, morphogenesis, NKX2-5 gene

## Introduction

Proper morphogenesis during the embryonic development is crucial for the heart to function effectively as a pump. The internal structure of the heart undergoes dramatic transformations over a short period, with remodeling continuing into the neonatal period. Following cardiac looping, endocardial cells in the outflow tract and atrioventricular canal regions undergo endothelial-to-mesenchymal transformation (EndoMT) to form cushion mesenchyme that eventually remodel into cardiac valves and septum. Recent studies have expanded this understanding, demonstrating that endocardial cells also undergo endothelial-to-hematopoietic transformation (EHT), contributing to cushion remodeling (Nakano et al., 2013; Shigeta et al., 2019; Liu et al., 2023b). These findings challenge the conventional view that embryonic hematopoiesis occurs exclusively in the yolk sac and aorta-gonad-mesonephros (AGM) region in mammals. This opinion article summarizes existing research on endocardial hematopoiesis and its role in cardiac morphogenesis.

In *Drosophila* embryos, hematopoiesis is closely linked to heart development. Both the heart and hematopoietic systems share developmental origins and molecular mechanisms, including Tinman (the orthologue of Nkx2-5), GATA factors, and Notch signaling (Mandal et al., 2004; Han and Olson, 2005). Specifically, the dorsal vessel, which serves as the heart tube in flies, is also integral to the development of hemocytes, blood cells that play roles analogous to mammalian macrophages in immune responses and tissue maintenance (Lebestky et al., 2000).

Studies have shown that endocardial hematopoiesis observed in mouse embryos is conserved in *Drosophila*. In mice, hematopoietic cells derived from endocardial cells are produced in an Nkx2-5-dependent manner (Nakano et al., 2013; Liu et al., 2023b), with macrophages as the predominant cell lineage involved in cardiac cushion remodeling (Shigeta et al., 2019; Liu et al., 2023b). However, the notion that endocardial cells give rise to macrophages via *de novo* hematopoiesis remains controversial (Liu et al., 2022; Liu et al., 2023a). Despite these debates, endocardial hematopoiesis has also been observed in zebrafish (Gurung et al., 2024; Bornhorst et al., 2024), supporting its evolutionary conservation.

This article addresses the ongoing controversies surrounding endocardial hematopoiesis and explores potential directions for future research in endocardial hematopoiesis, aiming to advance our understanding of its role in cardiac development.

## Discovery of endocardial hematopoiesis

In early mammalian cardiac primordia, cardiac progenitor cells marked by *Flk1*, *Isl1*, and *Nkx2-5* differentiate into cardiomyocytes, smooth muscle cells, and endothelial/endocardial cells (Moretti et al., 2006). Researchers discovered that these progenitors also express hematopoietic transcription factors, including *Gata1*, *Lmo2*, *Runx1*, and *Tal1* (Masino et al., 2004). Despite this finding, the significance of hematopoietic signatures in cardiac progenitor cells remained unclear for many years. Interestingly, an earlier study identified hematopoietic-like cells in the endocardial layer of zebrafish (Al-Adhami and Kunz, 1977), suggesting a possible evolutionary link between hematopoiesis and cardiogenesis.

**FIGURE 1:**
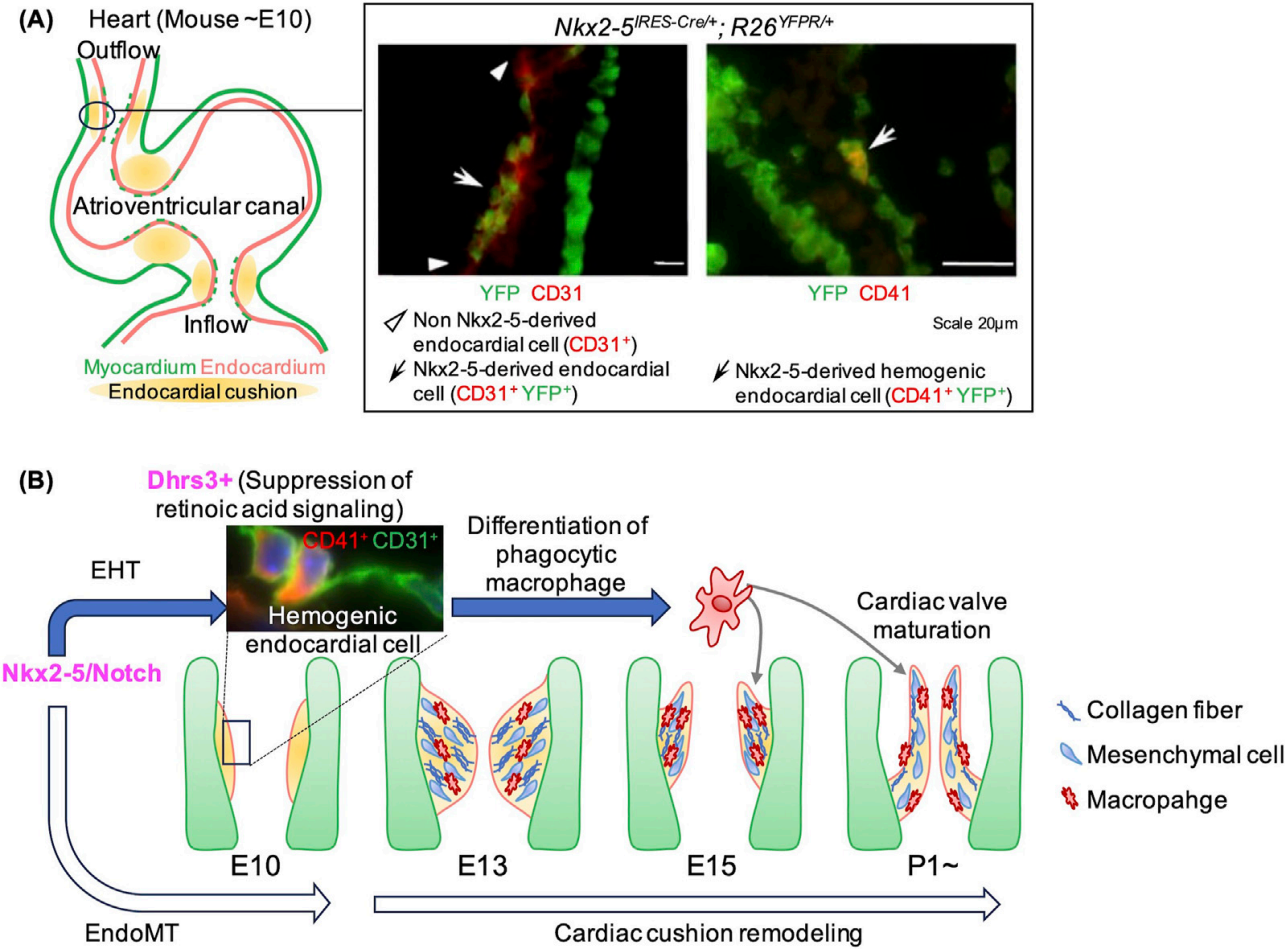
Endocardial hematopoiesis and its role in cardiac morphogenesis during mouse development. **(A)** Hemogenic endocardial cells are localized in regions of endocardial cushion formation and originate from the Nkx2-5 lineage. The immunofluorescent image depicts the outflow tract cushion in an *Nkx2-5* ^*IRES-CRE/+*^ *; R26* ^*YFPR/+*^ embryo, where Nkx2-5-derived cells are marked by YFP expression. Most ventricular cardiomyocytes and a subset of endocardial cells are positive for YFP (left). A subset of Nkx2-5-derived endocardial cells express CD41 (right). **(B)** The schematic illustrates our findings on the molecular mechanisms and the role of endocardial hematopoiesis. Nkx2-5/Notch signaling drive the transformation of endocardial cells into both mesenchymal cells (via EndoMT) and hematopoietic cells (via EHT). Hematopoietic cells derived from the endocardium express Dhrs3, which suppress retinoic acid signaling. This suppression promotes the differentiation of these cells into macrophages, which display enhanced phagocytic activity. These macrophages play a crucial role in remodeling the cardiac cushion, ultimately contributing to the formation of mature heart valves. Immunofluorescent staining images are adapted from Nakano et al. (2013).

While the plasticity of endocardial cells has primarily been studied in the context of their contributions to mesenchymal cells and coronary endothelial cells, their hematopoietic potential has remained unexplored (Gise and Pu, 2012; Nakano et al., 2016; Zhang et al.,2018; Tian et al., 2015; D’Amato et al., 2022). Endocardial cells, lining the inner surface of the heart, are typically squamous in shape. However, upon hematopoietic transformation, they adopt a rounded morphology and begin to express early hematopoietic markers such as CD41 and *Tal1* (Nakano et al., 2013; Collart et al., 2021). Our studies and others have identified endocardial cells expressing hematopoietic markers in the outflow tract, atrioventricular canal, and inflow tract of the mouse embryonic heart (Nakano et al., 2013; Yzaguirre and Speck, 2016). This localization pattern overlaps with distribution of *Nkx2-5* lineage endocardial cells and endocardial cushion (Figure 1A). *Nkx2-5* knockout (KO) mice die in mid-gestation due to lack of endocardial cushion formation and hypoplastic cardiomyocytes (Lyons et al., 1995; Tanaka et al., 1999). Notably, the KO mice also develop hematopoietic defects in yolk sac and endocardium (Lyons et al., 1995; Nakano et al., 2013). Therefore, Nkx2-5 is not only expressed in the hemogenic endocardial cells but also required for the hematopoiesis.

Using *in vivo* and single-cell RNA-sequencing (scRNA-seq) analysis (GSE76118 (Li et al., 2016)), we found that *Nkx2-5* KO mouse endocardium lacks both cushion endocardial cells and hematopoietic progenitor cells (Nakano et al., 2013; Liu et al., 2023b). This finding aligns with the tinman-dependent hematopoiesis observed in *Drosophila* larva (Mandal et al., 2004; Han and Olson, 2005), suggesting that *Nkx2-5*-dependent hematopoiesis is across species. Further scRNA-seq analysis revealed two key signaling pathways involved in *Nkx2-5*-dependent endocardial hematopoiesis: Notch signaling and retinoic acid (RA) signaling (Liu et al., 2023b). Forced activation of Notch signaling in *Nkx2-5*-lineage cells restored both endocardial cushion and hematopoietic cell deficits in *Nkx2-5*-null background, demonstrating that Notch signaling promotes endocardial cushion formation and hematopoiesis downstream of Nkx2-5. RA signaling also plays a critical role. *Dhrs3* (dehydrogenase/reductase 3) encoding an enzyme that reduces all-trans RA (atRA) levels was significantly downregulated in *Nkx2-5* KO endocardial cells. *Ex vivo* hematopoietic colony formation assays showed that excessive RA signaling inhibits hematopoietic progenitor differentiation, including macrophage differentiation, suggesting that RA suppression is essential for these processes. Forced activation of Notch signaling in *Nkx2-5*-lineage cells enhanced macrophage production with an increase in *Dhrs3*-positive proportions, linking the *Nkx2-5*-Notch signaling axis to *Dhrs3*-mediated RA regulation and macrophage differentiation (Liu et al., 2023b) (Figure 1B). It remains unclear whether *Nkx2-5* expression is directly required for EHT. As genome-wide ChIP-seq study has identified Nkx2-5 binding sites in the conserved regulatory regions of *Notch1*, *Jag1*, *Rbpjk*, and *Runx1*, some of these established regulators may be direct target of *Nkx2-5* (He and Pu, 2010). Further studies are required for establishing the precise mechanism of *Nkx2-5*-dependent hematopoiesis and EHT.

Flow cytometric analysis using *Nfatc1*-lineage tracing revealed that a small fraction of endocardial-derived tissue macrophages (2.6%–17.4%) persists in fetal hearts and into adulthood (Shigeta et al., 2019). However, the hemogenic activity of endocardial cells remains controversial. Two studies from Dr. Zhou’s group identified *Nfatc1*-labeled cells in yolk sac and failed to confirm hemogenic activity in mammalian endocardial cells (Liu et al., 2022; Liu et al., 2023a). These issues and implications are discussed elsewhere (Nakano and Liu, 2023).

Recently, live imaging studies in zebrafish have provided new insights into endocardial hematopoiesis. Gurung et al. observed EHT of endocardial cells as early as 24 h post-fertilization (hpf), corresponding to mouse E8.0, before the onset of heartbeat (Gurung et al., 2024). This process depends on *gata5/6* and *hedgehog* signaling rather than canonical hematopoietic transcription factors like *etv2/etsrp* and *scl/tal1*, with neutrophils as the primary outcome (Gurung et al., 22024). On the other hand, Bornhorst et al. reported increased endocardial hematopoiesis starting at 74 hpf, corresponding to mouse E10.5, when endocardial cushion formation is more advanced (Bornhorst et al., 2024). Their study utilized live imaging, lineage-tracing, and scRNA-seq analysis with a photoconversion-based approach to demonstrate that hemogenic endocardial cells give rise to hematopoietic stem/progenitor cells (HSPCs) by maintaining their adhesion to the endocardium via itga4 and vcam1 (Bornhorst et al., 2024). Together, these findings suggest that endocardial cells may also influence systemic hematopoiesis by serving as a source of neutrophils and an HSPC niche.

Further investigations using advanced live imaging and more sophisticated tracing techniques are needed to resolve ongoing controversies and clarify the contribution of hematopoietic endocardium to cardiac development and systemic hematopoiesis.

## Physiological significance of endocardial hematopoiesis

The physiological relevance of endocardial hematopoiesis is an emerging area of study. We have demonstrated that hematopoietic cells derived from endocardial cells differentiate into tissue macrophages that reside within the cardiac cushion mesenchyme (Nakano et al., 2013; Shigeta et al., 2019; Liu et al., 2023b). Bulk RNA-seq analysis revealed that endocardial macrophages are enriched in genes involved in antigen presentation, lysosome activity, and phagosomes function as compared with other tissue macrophage populations (Shigeta et al., 2019). Functional phagocytosis assays corroborated with these findings highlighting these macrophages’ phagocytic capabilities (Shigeta et al., 2019). To further elucidate the physiological role of endocardial-derived macrophages, we genetically ablated these cells by crossing *Nfatc1-cre* or *Nkx2-5-cre* mice with *Csf1r-flox/flox* mice, where the colony-stimulating factor 1 receptor (*Csf1r*), crucial for macrophage differentiation, was deleted specifically in the endocardium. Endocardial-derived macrophage-depleted mice exhibited cardiac valve anomalies characterized by excessive extracellular matrix (ECM) accumulation and increased cellularity. These findings indicate that endocardial-derived macrophages play a crucial role in proper valve remodeling (Shigeta et al., 2019; Liu et al., 2023b). Notably, despite the compensation of the total number of macrophages by the compensatory recruitment of monocyte-derived macrophages, the cardiac valve phenotypes persisted. This highlights that their unique, non-redundant role in cardiac cushion remodeling and valve formation (Figure 1B).

As discussed earlier, studies in zebrafish have reported distinct lineage contributions of endocardial-derived hematopoietic cells: At 24 hpf, Gurung et al. observed that hematopoietic cells detach from the endocardium and express neutrophil markers following EHT, suggesting that endocardial-derived cells may serve as a major source of neutrophils during early development (Gurung, et al., 2024). In contrast, at 74 hpf and later stages, Bornhorst et al. found that *de novo* EHT in the endocardium maintain cell adhesion to the endocardial layer while differentiating into HSPCs (Bornhorst et al., 2024). Unlike our findings in mice, both zebrafish studies reported minimal contributions of endocardial-derived cells to macrophage populations in cardiac valves. This discrepancy likely reflects differences in developmental stages and species-specific physiological requirements. Endocardial cushion remodeling is not as extensive in zebrafish valve formation. Zebrafish valve mesenchyme cells form valve cusps that are thin and simple in structure (Pestel et al., 2016; Gunawan et al., 2020), whereas mammalian valve formation involves extensive ECM remodeling and sculpting to generate structurally complex and durable valves [Reviewed in (MacGrogan et al., 2014; O’Donnell and Yutzey, 2020)].

These distinctions underscore the unique and indispensable role of endocardial-derived macrophages in mammalian cardiac development, particularly in the context of the more intricate and mechanically demanding architecture of mammalian valves. Their specialized functions in remodeling the cardiac cushion mesenchyme are vital for ensuring proper valve formation, highlighting their evolutionary significance in adapting to the higher mechanical stresses of the mammalian circulatory system.

## Discussion

Our studies demonstrate that endocardial cells undergo both EHT and EndoMT in an *Nkx2-5*/Notch-dependent manner. These processes generate hematopoietic cells that differentiate into macrophages through the inhibition of RA signaling. These results reveal a previously underexplored role of endocardial hematopoiesis in local tissue remodeling during heart development. However, significant questions remain. The ultimate fate of endocardial-derived hematopoietic cells, such as their potential contributions to other hematopoietic lineages or their broader roles in cardiac or systemic physiology, is still unclear. Additionally, the mechanisms that govern the balance between EHT and EndoMT in these cells and their interactions with other macrophage populations warrant further investigation. Addressing these gaps will be crucial for a comprehensive understanding of endocardial hematopoiesis and its implications for cardiovascular development and homeostasis.

Overcoming current technical limitations, such as live imaging of these rare cell populations and their dynamic transitions, will be essential for advancing our understanding of endocardial hematopoiesis. Advanced methodologies, including single-cell multiomics and cutting-edge lineage-tracing approaches, hold the potential to unravel their developmental trajectories and physiological significance. Future studies aimed at addressing these questions will provide critical insights into the unique contributions of endocardial hematopoiesis in heart development and its potential relevance to other organ systems. Such knowledge could have profound implications for understanding both normal physiology and disease processes across multiple biological contexts.
